# *Erratum*: Vol. 65, No. 52

**DOI:** 10.15585/mmwr.mm6602a10

**Published:** 2017-01-20

In the report “Zika Virus —10 Public Health Achievements in 2016 and Future Priorities,” on page 1482, the first sentence under the heading “3. Identifying Sexual Transmission of Zika Virus Infection,” should have read “In late January, CDC, in collaboration with Texas health officials, worked to confirm sexual contact as the source of Zika virus infection in a Dallas man whose partner had traveled to Venezuela (*14*) and issued guidance for the prevention of sexual trans­mission of Zika virus in February (*15*).”

